# Strengthening early relational health: strategies and system-level considerations for pediatric primary care

**DOI:** 10.3389/fped.2026.1773528

**Published:** 2026-06-05

**Authors:** Sarah Behrens, Brenda Salley

**Affiliations:** Department of Pediatrics, Division of Developmental and Behavioral Sciences, University of Kansas Medical Center, Kansas City, KS, United States

**Keywords:** caregiver-child relationships, early childhood development, early relational health, pediatric primary care, social-emotional development

## Abstract

Early relational health (ERH) in the earliest years of life represents a critical foundation for children's social-emotional development, learning, and lifelong well-being. Safe, responsive caregiver–child relationships shape brain architecture, regulate stress physiology, and buffer adversity. Growing recognition of this relational foundation has led the American Academy of Pediatrics (AAP) to call for pediatric primary care to more systematically support ERH. Yet system-level barriers, including limited visit time, insufficient training, and fragmented referral pathways, and inadequate reimbursement, constrain the field's ability to address relational and social-emotional needs universally. This *Perspective* synthesizes emerging evidence and highlights practical strategies for integrating ERH promotion into pediatric primary care. Approaches such as brief caregiver coaching, strengths-based observation, social-emotional screening, and thoughtfully designed digital supports, implemented by interdisciplinary teams and linked to community-based services, can strengthen family capacity and enhance developmental trajectories. Advancing ERH in pediatric care will require coordinated practice transformation, cross-sector collaboration, and policy reforms that enable scalable, relationship-centered models of care.

## Introduction

The earliest years of life are a period of rapid neurodevelopment when infants and young children build the foundations for early relational health (ERH) – attuned, responsive relationships with caregivers that shapes social-emotional development, learning, and life-long well-being (1, 2). ERH supports children's ability to experience and regulate emotions, form secure attachments, and explore their environment within supportive caregiving contexts. ERH is the relational foundation upon which social-emotional skills develop, supporting resilience and buffering against adversity (3, 4). Despite robust evidence linking ERH to long-term developmental outcomes, pediatric primary care has not fully leveraged its potential, constrained by systemic barriers, including time limits, workforce training gaps, and reimbursement structures (5).

Safe, responsive caregiver-child relationships shape brain architecture, calibrate stress response systems, and establish patterns for future learning, behavior, and health. When caregiving relationships are disrupted by chronic stress, instability, poverty, racism, and other structural inequities, children face increased risk for difficulties in emotional regulation, behavior, and developmental functioning, contributing to widening disparities across childhood (6–8). Conversely, nurturing and consistent relationships buffer the effects of toxic stress and support healthy developmental trajectories, even in the presence of adversity (9, 10).

Reflecting this evidence, the American Academy of Pediatrics (AAP) identifies ERH as a core component of healthy development and positions pediatric primary care as a universal platform for fostering it (11). The AAP's ERH initiative emphasizes everyday relational moments, strengths-based engagement, and culturally responsive guidance as essential elements of well-child care. Through repeated contact with families during early childhood, pediatric primary care offers unique opportunities to reinforce responsive caregiving, support early social-emotional development, and identify emerging concerns before they escalate (5).

Despite its central role in development, ERH promotion has not yet been systematically integrated into routine well-child visits (5). System-level constraints – including limited visit time, insufficient training, competing clinical demands, lack of brief, workflow-compatible relational strategies, and reimbursement structures that undervalue preventive relational care – restrict the consistent delivery of ERH-informed supports (11–13). Within these constraints, pediatric teams already provide meaningful developmental guidance and respond to behavioral and relational concerns as they arise. With greater system alignment and accessible, evidence-based strategies, pediatric teams could more effectively address early signs of emotional dysregulation, strengthen caregiver–child relationships, and mitigate downstream challenges such as early childhood expulsion, school exclusion, and chronic mental health conditions (14–16).

This Perspective shifts the focus from whether ERH should be integrated into pediatric primary care t how it can be implemented, integrated and scaled within real-world clinical systems. Although a growing body of evidence supports brief, relationship-centered interventions delivered in primary care, most models have been evaluated in isolation and demonstrate modest effects. Less is known about how complementary ERH strategies can be layered within routine workflows, supported by clinical care teams, and sustained through cross-sector partnerships. We argue that advancing ERH requires coordinated efforts across systems, including practical strategies that fit within brief clinical encounters, workforce training and coaching, and policies and payment models that incentivize prevention and relationship-centered care (5, 11, 17, 18).

Pediatric primary care provides a uniquely scalable platform for advancing ERH due to its near-universal reach, longitudinal relationships with families, and trusted role in early childhood. These features position pediatric settings to deliver brief, relationship-centered supports, identify emerging concerns and connect families to community-based resources. When implemented consistently, ERH-aligned practices can strengthen caregiver capacity, support children's developing self-regulation and social competence, and buffer the effects of adversity across the life course. However, realizing this potential requires approaches that fit within time-constrained visits, leverage interdisciplinary teams, and align with existing clinical workflows; without this alignment, ERH efforts remain inconsistent and difficult to sustain at scale (5).

## Discussion

### Components of ERH-aligned care in pediatric primary care

To maximize pediatric primary care as a vehicle for promoting ERH and child development, practices must implement evidence-based strategies, strengthen workforce capacity, and advance policies that incentivize prevention and team-based care (5, 14).

#### Screening and risk identification

Routine screening for social-emotional, behavioral concerns, and social determinants of health provides a foundation for early identification and tailored support. While validated tools are increasingly available, integrating them into brief visits, ensuring follow-up, and building team confidence remain challenges (5, 11, 19). Embedding developmental specialists, providing team-based training, and leveraging collaborative networks can enhance early detection, facilitate proactive support, and strengthen family capacity. Centralized service navigation systems such as Help Me Grow and FIND, demonstrate how coordinated referral pathways can connect families efficiently and equitably to community resources and support timely follow-up (5, 13, 20).

#### Brief relationship-centered supports embedded in care

A growing set of pediatric interventions demonstrate that brief, relationship-centered supports can be embedded in well-child care (5). These programs typically include caregiver coaching, strengths-based observations, and guidance that reinforces responsive interactions in everyday routines. Programs such as HealthySteps, Reach Out and Read, and PlayReadVIP (formerly the Video Interaction Project) illustrate different delivery models, but share a common emphasis on brief, repeatable interactions in coordination with primary care (21, 22).

Although many of these programs have demonstrated meaningful improvements in caregiver behaviors and child outcomes, much of the existing evidence reflects evaluations of individual interventions implemented in isolation within pediatric settings (23). Effect sizes for single programs are often modest, reflecting the complexity of relational and developmental processes as well as the multiple contextual stressors families face (5, 9). Emerging implementation efforts suggest that combining complementary strategies such as developmental screening, caregiver coaching, behavioral health consultations, and coordinated referral pathways may strengthen impact by addressing multiple dimensions of ERH simultaneously (5, 24, 25). However, research examining how layered ERH strategies function together within pediatric workflows and how integrated models can be sustainably scaled across health systems remains limited and represents an important area for future study.

Evidence from early care and education and other child-serving systems (e.g., childcare programs, early intervention, home visiting, community mental health services) further highlights the value of brief, consistent relational strategies delivered within everyday routines. Translating these principles into pediatric practice requires intentional adaptation, workforce training, and alignment with the realities of well-child care delivery. Collaborating across these sectors can extend the reach of relationship-centered interventions, strengthen family capacity, and support positive social-emotional and behavioral outcomes in children, while maintaining the medical home as the primary locus for preventive guidance (6, 39).

Emerging pediatric models illustrate how team-based approaches can strengthen ERH within the medical home. For example, Talk With Me Baby (TWMB), a language promotion intervention embedded within routine well-child care, has been shown to be both acceptable and feasible for pediatric care teams to deliver, and preliminary data indicates increases in parent language-building interactions that support early communication and relational engagement (26). Models such as DULCE (Developmental Understanding and Legal Collaboration for Everyone) further demonstrate how team-based approaches that integrate developmental support, legal screening, and care coordination can address family stressors and promote ERH within pediatric primary care (27, 28). Together, these programs highlight the feasibility of embedding preventive, relational-centered supports within pediatric primary care and demonstrate the potential for broader integration of ERH strategies within routine well-child care.

#### Cross-sector collaboration

Integrating behavioral health specialists and care coordinators into pediatric teams is essential, as pediatricians alone cannot address the complex, multifaceted social-emotional needs of children and families (5, 11). These professionals provide coaching, screening, and follow-up support, helping families navigate resources and reinforcing ERH between visits. HealthySteps and DULCE embed family specialists within the medical home, exemplifying practical, team-based models (27, 40). Strong connections with community-based programs (i.e., early intervention, home visiting, community mental health services) further ensure continuity of care. Embedding pediatric interventions within broader community networks including home visiting, early intervention, and maternal mental health services, can amplify impact, increase effect sizes, and support more comprehensive ERH outcomes by addressing multiple dimensions of family and child well-being simultaneously [(5, 6); Chudzik & Corr, 2025]. Research with home visitors demonstrates active engagement in developmental monitoring, while identifying barriers such as cultural-linguistic challenges and screening tool implementation (29). Structured collaboration, warm handoffs, and co-located services prevent gaps, reinforce developmental gains, and reduce the risk of exclusionary discipline, advancing equity and resilience for children and families (16, 30).

#### Policy and payment advocacy

Sustaining and scaling relationship-centered supports requires policies and payment models that value preventive, team-based care. Current fee-for-service structures often fail to reimburse time spent on screening, coaching, and care coordination, including the absence of billing pathways for ERH care, limited coverage for preventive services without a diagnosis, and low reimbursement rates, all of which constrain routine ERH practices despite strong evidence of effectiveness (5, 9, 14). Capitated or value-based models can partially mitigate these barriers by incentivizing prevention and population-level outcomes, but even these approaches often lack specific metrics or funding to support relational health interventions within well-child care. Addressing these gaps through targeted payment strategies is critical to enable consistent delivery of ERH-aligned practices. Aligning Medicaid, CHIP, and other funding streams to incentivize workforce training, team-based coaching, and high-quality social-emotional development promotion can support equitable access and broader adoption (5). Programs such as HealthySteps and Help Me Grow demonstrate that policy-aligned funding, when paired with workflow-adapted interventions, can facilitate sustainable scaling of ERH supports while improving equity and developmental outcomes (15, 31, 40).

Across ERH models, a consistent pattern emerges: pediatric practices can implement individual ERH-aligned strategies, but struggle to integrate and sustain multiple approaches as a coherent system of care. These challenges are not primarily due to lack of effective interventions, but to misalignment across payment and policy, clinical workflow, and cross-sector coordination (5). As a result, ERH efforts often remain fragmented, limiting their cumulative impact and scalability.

### Future directions: advancing early relational health in pediatric primary care

Strengthening ERH within pediatric primary care requires evidence that is both rigorous and practice-ready, alongside sustainable infrastructure and workforce capacity (5). In line with the AAP's call to prioritize relational health in routine well-child care, we outline four interrelated domains that represent key priorities for research, implementation, and policy innovation.

#### Evidence generation and implementation science

Although momentum is growing, clear evidence is still needed on how to successfully integrate and maintain ERH strategies - such as social-emotional screening, brief caregiver coaching, and strengths-based feedback - into fast-paced pediatric workflows (5). Existing models, including HealthySteps and integrated behavioral health programs, demonstrate both the acceptability and feasibility of delivering these strategies within routine visits (21, 27). While these models provide strong proof-of-concept, most evidence reflects implementation of single programs (5). Research is still limited on how layered or integrated ERH strategies function together within pediatric workflows, and how they can be scaled across diverse health systems to enhance overall impact. Future studies should test scalable models across diverse clinical environments, including federally qualified health centers, hospital clinics, and private practices to understand barriers, facilitators, and contextual adaptation needed for broad implementation (5, 32–34). Implementation science can accelerate progress by identifying the mechanisms through which strategies enhance caregiver-child relationships, determining when and how adaptations strengthen impact, and examining contextual factors that influence adoption, fidelity, and reach (5, 24, 25). Priorities include pragmatic trials, hybrid effectiveness-implementation designs, and mixed-methods approaches that center feasibility, cultural responsiveness, and real-world utility.

#### Sustainable infrastructure and financing

Scaling ERH supports requires infrastructure that enables team-based, preventive care. Research should evaluate workflow designs, staffing models, and technology supports, including EHR-integrated prompts, telehealth-delivered coaching, and automated digital screening, that streamline implementation while preserving relational quality (5, 35, 38). Financial sustainability is also essential. Fee-for-service structures often undervalue prevention and relational work, limiting the reach of ERH interventions (5). Innovative financing strategies, such as Medicaid reimbursement for dyadic care, value-based payment models that reward developmental promotion, and state-level incentives for integrated behavioral health, can help close this gap. Long-term progress requires coordinated alignment across pediatric, mental health, and early childhood systems to sustain integrated ERH strategies, ensure consistent delivery, and enable systematic scaling beyond individual programs (5).

#### Strengths-based design and community partnership

ERH emerges within responsive relationships, yet families access to supports varies across social, geographic, and structural contexts (5). Pediatric models should expand delivery pathways so supports reach families where they already spend time, including pediatric clinics, home visiting, and digital platforms (e.g., mobile coaching, text-based prompts, interactive caregiver resources) (5). Programs that are culturally responsive, linguistically accessible, and co-designed with families can reinforce relational strengths and trust (9). Lessons from early care and education and other child-serving systems highlight the value of brief, consistent relational strategies delivered within everyday routines, which can inform adaptation in pediatric practice. Integrating pediatric interventions with community-based services such as home visiting, early interventions, and maternal mental health programs can amplify effects on relational health by addressing multiple domains of family and child well-being concurrently (5). Digital innovations such as video modeling, app-based supports, and automated feedback can extend ERH guidance between visits while maintaining relational focus (16).

#### Workforce development and cross-sector alignment

A relationally attuned workforce is central to strengthening ERH in pediatric care (5). Training in early brain development, stress physiology, relational health, and trauma-informed practice should begin in medical school and continue across residency and ongoing professional development (5, 9). Education should also build skills in reflective practice, family-centered communication, and understanding the systemic factors that shape caregiving (41). Providers benefit from brief, practical coaching scripts, observation prompts, family handouts, and digital resources that allow integration of relational support without increasing burden – a finding supported by syntheses showing that pediatric primary care parenting education interventions can be delivered feasibly with supportive materials and tools (36). Workforce development should prepare clinicians to deliver coordinated, multi-component ERH strategies that integrate seamlessly across clinical and community settings (5). Because families receive relational support across multiple systems, sustained impact requires alignment among pediatric teams, early intervention, home visiting, and community mental health services that share language, coordinate care, and create warm handoffs (5, 37).

Through advances in clinical practice, implementation science, cross-sector partnership, and workforce preparation, pediatric primary care can evolve into a fully relationally centered system, in which integrated, scalable ERH strategies are embedded as a core purpose of well-child care.

## Conclusion

Promoting ERH within pediatric primary care is essential to nurturing healthy, resilient children and strengthening the relational foundations that support lifelong learning, behavior, and well-being. Early caregiving relationships shape brain development, regulate stress-response systems, and establish the conditions in which children develop social-emotional competence, secure attachment, and adaptive coping. When relationally attuned practices such as routine social-emotional screening, brief caregiver coaching, strengths-based observation, and coordinated cross-sector supports are integrated into everyday well-child care, pediatric settings become powerful and accessible platforms for fostering connection, regulation, and resilience.

Achieving this represents a paradigm shift that requires coordinated action across clinical practice, workforce development, financing, and policy. Sustaining progress will depend on continued investment in evidence-based, culturally responsive models and a workforce prepared to deliver developmentally informed, relationship-centered care. With aligned systems, strong community partnerships, and practical strategies, including brief relational-health coaching and thoughtfully designed digital supports that fit within real-world workflows, pediatric primary care can fully realize its role in advancing ERH. By centering relationships in early health care, we strengthen family capacity, improve developmental trajectories, and help build communities where all young children have the opportunity to thrive.

## Data Availability

The original contributions presented in the study are included in the article/Supplementary Material, further inquiries can be directed to the corresponding author.
